# Relationship between serum bilirubin concentrations and diabetic nephropathy in Shanghai Han’s patients with type 1 diabetes mellitus

**DOI:** 10.1186/s12882-017-0531-8

**Published:** 2017-03-31

**Authors:** Xu Li, Lei Zhang, Haibing Chen, Kaifeng Guo, Haoyong Yu, Jian Zhou, Ming Li, Qing Li, Lianxi Li, Jun Yin, Fang Liu, Yuqian Bao, Junfeng Han, Weiping Jia

**Affiliations:** Department of Endocrinology and Metabolism, Shanghai Jiao Tong University Affiliated Sixth People’s Hospital, Shanghai Clinical Center for Diabetes, Shanghai Diabetes Institute, Shanghai Key Laboratory of Diabetes Mellitus, Shanghai Key Clinical Center for Metabolic Disease, 600Yishan road, Shanghai, 200233 China

**Keywords:** Type 1 diabetes mellitus, Diabetic nephropathy, Bilirubin concentrations

## Abstract

**Background:**

Recent studies highlight a negative association between total bilirubin concentrations and albuminuria in patients with type 2 diabetes mellitus. Our study evaluated the relationship between bilirubin concentrations and the prevalence of diabetic nephropathy (DN) in Chinese patients with type 1 diabetes mellitus (T1DM).

**Methods:**

A total of 258 patients with T1DM were recruited and bilirubin concentrations were compared between patients with or without diabetic nephropathy. Multiple stepwise regression analysis was used to examine the relationship between bilirubin concentrations and 24 h urinary microalbumin. Binary logistic regression analysis was performed to assess independent risk factors for diabetic nephropathy. Participants were divided into four groups according to the quartile of total bilirubin concentrations (Q1, 0.20–0.60; Q2, 0.60–0.80; Q3, 0.80–1.00; Q4, 1.00–1.90 mg/dL) and the *chi*-square test was used to compare the prevalence of DN in patients with T1DM.

**Results:**

The median bilirubin level was 0.56 (interquartile: 0.43–0.68 mg/dL) in the DN group, significantly lower than in the non-DN group (0.70 [interquartile: 0.58–0.89 mg/dL], *P* < 0.001). Spearman’s correlational analysis showed bilirubin concentrations were inversely correlated with 24 h urinary microalbumin (*r* = -0.13, *P* < 0.05) and multiple stepwise regression analysis showed bilirubin concentrations were independently associated with 24 h urinary microalbumin. In logistic regression analysis, bilirubin concentrations were significantly inversely associated with nephropathy. In addition, in stratified analysis, from the first to the fourth quartile group, increased bilirubin concentrations were associated with decreased prevalence of DN from 21.90% to 2.00%.

**Conclusion:**

High bilirubin concentrations are independently and negatively associated with albuminuria and the prevalence of DN in patients with T1DM.

## Background

Diabetic nephropathy (DN) is the most common cause of end-stage renal disease worldwide, which remains a major cause of morbidity and mortality in patients with T1DM [1]. Oxidative stress may be a common pathway linking diverse, seemingly distinct, potential mechanisms underlying the pathogenesis of complications in diabetes, including nephropathy [2]. Bilirubin is the end product of haem catabolism and it acts as a powerful biological antioxidant [3, 4]. A study on Gilbert syndrome (GS) reported that the prevalence of ischemic heart disease (IHD) was 2% in GS patients (who are characterised by high bilirubin concentrations) compared to 12.1% in the general population, indicating that chronic hyperbilirubinemia prevents the development of IHD by increasing the antioxidant capacity of serum [5]. In a recent study that used the deoxycorticosterone acetate (DOCA)-salt model of hypertension in Heine oxygenase (HO)-1^-^/^-^ and HO-1^+^/^+^ mice, systolic arterial pressure was significantly elevated in HO-1^-^/^-^ mice treated with DOCA salt but not in HO-1^+^/^+^ mice; in addition, DOCA-salt impaired vasorelaxation was noted in wild-type rats but not in hyperbilirubinemic rats [6]. These results suggest that the HO-1 isozyme and the product bilirubin may have protective effects on vascular disease. This finding has also been confirmed in a model of balloon injury [7]. In addition, diabetic hyperbilirubinemic Gunn j/j rats excrete significantly less urinary albumin than diabetic non-hyperbilirubinemic Gunn j/+ rats and administration of biliverdin (5 mg/kg) protects against both albuminuria and renal mesangial expansion in db/db mice [8]. These findings suggest that bilirubin and biliverdin may protect against DN. A population-based study showed that high bilirubin concentrations in serum are associated with reduced risk of DN [9]. Furthermore, several longitudinal studies on healthy subjects and type 2 diabetes mellitus (T2DM) also demonstrated that low serum bilirubin concentration could be a novel risk factor for the development of albuminuria in patients with type 2 diabetes [10, 11]. However, renal disease remains a major cause of morbidity and mortality in patients with T1DM and the association between bilirubin concentrations in serum and the prevalence of diabetic nephropathy in patients with T1DM has not yet been studied. Therefore, in the present study, we evaluated the association between bilirubin concentrations in serum and the prevalence of DN, and we hypothesized that bilirubin concentrations may inversely associate with the prevalence of DN in Chinese patients with T1DM.

## Methods

### Study population

This was a cross-sectional, population-based study involving 258 patients with T1DM. Participants were hospitalised patients who presented at the Department of Endocrinology and Metabolism, Shanghai Jiaotong University Affiliated Sixth People’s Hospital between January 2008 and January 2013. T1DM was defined as anti-glutamate decarboxylase (GAD) antibody level ≥ 1.5 U/mL and injecting insulin at least three times daily or using an insulin pump, and DN were diagnosed according to KDOQI Clinical Practice Guidelines and Clinical Practice Recommendations for Diabetes and Chronic Kidney Disease for 2007 [12]. Hypertension was diagnosed if the patient had a blood pressure greater than 140/90 mmHg or used anti-hypertensive drugs. Subjects were excluded for the following reasons: absence of data (bilirubin, haemoglobin A1c [HbA1c]), abnormal thyroid function (hyperthyroidism or hypothyroidism), elevated serum levels of creatinine (>124 μmol/L), serum bilirubin concentrations > 2.0 mg/dL, and T2DM or specific diabetes. In addition, patients were excluded if they had confounding hepatobiliary or haemolytic disease, hepatitis B or C, alcoholic liver disease, gallstones, cirrhosis, IgA nephropathy, or urinary tract infections.

### Clinical and laboratory measurements

The clinical parameters investigated included age, height, weight, duration of diabetes, systolic blood pressure (SBP), diastolic blood pressure (DBP), waist circumference (WC), hip circumference, and waist-to-hip ratio (WHR). Biochemical variables were analysed after an overnight fast of at least 10 h and included fasting plasma glucose (FPG), 2 h postprandial glucose (2hPG), glycated haemoglobin A1c (HbA1c), glycated albumin (GA), C-reactive protein (CRP), 30-min postprandial venous C peptide, 120-min postprandial venous C peptide, albumin, aspartate aminotransferase (AST), alamine aminstransferase (ALT), gamma glutamytransferase (γ-GT), total cholesterol (TC), triglyceride level (TG), low-density lipoprotein cholesterol (LDL-C), high-density lipoprotein cholesterol (HDL-C), haemoglobin (Hb), 24 h urinary microalbumin, total GFR, uric acid, creatinine (Cr), and total bilirubin.

Subject height was measured to the nearest 0.1 cm with subjects not wearing shoes and weight was measured to the nearest 0.1 kg while the subjects wore light clothing; BMI was calculated as weight (kg) divided by the square of the height (m). SBP and DBP were measured after participants had rested for at least 5 min. WC (cm) was measured midway between the costal margin and the iliac crest at the end of a normal expiration and the hip circumference was measured as the circumference around both greater femoral trochanters. The WHR was calculated by dividing waist by hip circumference (cm). All biochemical determinations were performed using the same standard laboratory methods. After overnight fasting, blood was drawn early in the morning from the antecubital vein into vacuum tubes to determine the concentrations of fasting plasma glucose (FPG), C peptide, CRP, and concentrations of lipid components and liver enzymes. Bilirubin concentrations from serum samples were determined using the vanadate oxidation method.

Measurement of GFR using the ^99m^Tc-diethylene triamine pentaacetic acid (^99m^Tc-DTPA) renal dynamic imaging method, and ^99m^Tc-DTPA renal dynamic imaging (modified Gate’s method) was measured by Millennium TMMPR SPECT from General Electric Medical System [13]. Urine albumin excretions were evaluated by calculating total amounts of 24 h urinary microalbumin. The stage of albuminuria was defined as normal if no more than 30 mg/24 h, microalbuminuria if 24 h urinary microalbumin was 30–299 mg/24 h, and macroalbuminuria if 24 h urinary microalbumin was equal to or higher than 300 mg/24 h [12].

### Statistical analysis

Data are expressed as the means + standard deviation (SD) for a normal distribution of variables or as the median (interquartile range) for a skewed distribution of variables. For continuous data with a normal distribution and a skewed distribution between DN and non-DN patients, unpaired Student’s *t*-tests and nonparametric tests, respectively, were used for statistical analyses. Categorical variables were compared using a *chi*-square test. Spearman’s correlational analysis between 24 h urinary microalbumin, and other variables was performed. Multiple stepwise regression analysis was performed to assess the relationship between bilirubin concentrations and 24 h urinary microalbumin. Binary logistic regression analysis was performed to assess independent risk factors for diabetic nephropathy. Participants were divided into four groups according to the quartile of total bilirubin concentrations and the *chi*-square test was used to compare the prevalence of DN in patients with T1DM. The first quartile was 0.20–0.60 mg/dL, second quartile was 0.60–0.80 mg/dL, third quartile was 0.80–1.00 mg/dL, and fourth quartile was 1.00–1.90 mg/dL. All statistical analyses were performed using SPSS version 17.0 for Windows (SPSS, Chicago, IL, USA). A *P*-value less than 0.05 was considered to indicate statistical significance.

## Results

### Patient demographics and laboratory data

This study included 33 DN and 225 non-DN patients with T1DM. The general and biochemical characteristics of the DN patients (16 males and 17 females) and non-DN patients (111 males and 114 females) are shown in Table 1. The prevalence of patients with diabetic nephropathy, without nephropathy, were 12.8% (*n* = 33), 87.2% (*n* = 225), respectively. Of 33 patients with diabetic nephropathy, 20 were with microalbuminuria and 13 were with macroalbuminuria. The median age was 54 years (34.50–66 years) in DN subjects and 49 years (31–60 years) in non-DN patients. Median bilirubin concentrations were 0.56 mg/dL (0.43–0.68 mg/dL) in the DN group and 0.70 mg/dL (0.58–0.89 mg/dL) in non-DN patients (*P* < 0.01), a significant difference. 24 h urinary microalbumin level was significantly higher in the DN group than the non-DN group (196.67 mg vs. 7.01 mg, *P* < 0.01) and the same trend was observed with the duration of diabetes, SBP, WHR, uric acid, creatinine levels (*P* < 0.01), whereas albumin, haemoglobin, and total GFR levels were lower in the DN group (*P* < 0.05).

**Table 1:** Patient demographics and laboratory data

	DN (*n* = 33)	Non-DN (*n* = 225)	*P*-value
Male: Female	16:17	111:114	0.98
Age (years)	54 (34.50–66)	49 (31–60)	0.06
Duration of diabetes (years)	13.92 (5.50–20)	4.56 (1–8)	<0.001^**^
BMI (kg/m^2^)	22.67 (21.85–22.75)	21.85 (17.75–33.65)	0.09
SBP (mmHg)	130 (120–140)	120 (110–125)	<0.001^**^
DBP (mmHg)	79 (70–80)	78 (70–80)	0.81
WHR	0.87 (0.85–0.94)	0.86 (0.83–0.88)	0.01^*^
FPG (mmol/L)	8.24 (5.71–11.47)	7.73 (6.18–10.70)	0.48
2hPG (mmol/L)	12.74 + 6.73	14.03 + 5.57	0.23
HbA1c (mmol/mol)	79 (62–95)	79 (60–105)	0.68
GA (%)	27.50 (21.50–31)	27.50 (23–34)	0.44
CRP (mg/L)	1.01 (0.66–3.87)	0.68 (0.27–2.86)	0.25
Uric acid (mg/dL)	328 (246.50–414.5)	272 (223–316.50)	<0.001^**^
Cr (mg/dL)	78 (60–99.50)	63 (52–74)	0.01^*^
Fasting C peptide (ng/mL)	0.19 (0.03–1.13)	0.29 (0.05–0.80)	0.56
30-min postprandial venous C peptide (ng/mL)	0.1 (0.03–0.96)	0.35 (0.05–1.17)	0.93
120-min postprandial venous C peptide (ng/mL)	0.08 (0.03–1.62)	0.48 (0.07–1.58)	0.64
Albumin (g/dL)	41 (37.50–43)	42.44 (40–45)	<0.001^**^
ALT (U/L)	16 (11–24.5)	17 (12–25)	0.56
AST (U/L)	18 (15–24)	19 (15–24)	0.42
γ-GT(U/L)	18 (16–23.50)	15 (12–22)	0.73
Total Bilirubin (mg/dL)	0.56 (0.43–0.68)	0.70 (0.58–0.89)	<0.001^**^
TC (mmol/L)	4.75 + 1.34	4.41 + 0.99	0.09
TG (mmol/L)	1.09 (0.87–1.62)	0.88 (0.70–1.17)	0.07
HDL-C (mmol/L)	1.28 (0.95–1.66)	1.27 (1.10–1.60)	0.59
LDL-C (mmol/L)	2.69 (2.18–3.36)	2.68 (2.16–3.39)	0.17
Haemoglobin (g/dL)	126 (109.50–136.5)	130 (74–171)	0.04^*^
24 h urinary microalbumin (mg/24 h)	196.67(49.27–1123.33)	7.01 (4.89–10.44)	<0.001^**^
Total GFR (mL/min)	81.75 (60.35–96.03)	98.95(86.95–107.20)	<0.001^**^

Data are expressed as the means + standard deviation (SD) for normal distribution variables or as the median (interquartile range) for skewed distribution variables

*Abbreviations*: *DN* diabetic nephropathy, *T1DM* type 1 diabetes mellitus, *BMI* body mass index, *SBP* systolic blood pressure, *DBP* diastolic blood pressure, *WHR* waist-to-hip ratio, *FPG* fasting plasma glucose, *2hPG* 2 h postprandial glucose, *HbA1c* glycated haemoglobin A1c, *GA* glycated albumin, *Cr* creatinine, *CRP* C-reactive protein, *ALT* alanine aminotransferase, *AST* asparatate aminotransferase, γ*-GT* γ-glutamyltranspeptidase, *TC* total cholesterol, *TG* triglycerides, *HDL-C* high-density lipoprotein cholesterol, *LDL-C* low-density lipoprotein cholesterol, *GFR* glomerular filtration rate, *OR* odds ratio, *95% CI* 95% confidence interval

^*^ *P* < 0.05, ^**^ *P* < 0.01

### Bilirubin concentrations in serum are independently and negatively associated with 24 h urinary microalbumin

Previous studies have shown that microalbuminuria can indicate the progression of DN in patients with T1DM [14, 15]. To investigate the risk factors associated with microalbuminuria, we performed Spearman’s correlation analysis between bilirubin concentrations and other biochemical characteristics. As expected, 24 h urinary microalbumin was negatively correlated with bilirubin concentrations (*r* = -0.13, *P* < 0.05), HDL-C (*r* = -0.16, *P* = 0.01), and total GFR (*r* = -0.20, *P* < 0.001) and positively correlated with duration of diabetes, BMI, SBP, WHR, CRP, uric acid, Cr, TG levels (all *P* < 0.001). These results are presented in Table 2. To determine whether bilirubin concentrations were independently correlated with 24 h urinary microalbumin, we performed multiple stepwise regression analysis. Cr (β = 0.45, *P* < 0.001), total bilirubin (β = -0.19, *P* < 0.001), SBP (β = 0.15, *P* = 0.005) levels were independently related to 24 h urinary microalbumin. This result is shown in Table 3.

**Table 2:** Correlations of 24 h urinary microalbumin with bilirubin concentrations and other characteristics in subjects with T1DM

	24 h urinary microalbumin (unadjusted)	
	*r*	*P-*value
Age	0.07	0.24
Duration of diabetes	0.18	<0.001^**^
BMI	0.18	<0.001^**^
SBP	0.30	<0.001^**^
DBP	0.08	0.23
WHR	0.30	<0.001^**^
FPG	0.06	0.33
2hPG	− 0.10	0.13
HbA1c	0.02	0.72
GA	− 0.05	0.45
CRP	0.19	<0.001^**^
Uric acid	0.23	<0.001^**^
Cr	0.20	<0.001^**^
Fasting C peptide	0.01	0.92
30-min postprandial venous C peptide	− 0.02	0.80
120-min postprandial venous C peptide	− 0.02	0.74
TC	− 0.01	0.98
TG	0.24	<0.001^**^
HDL-C	− 0.16	0.01^*^
LDL-C	− 0.01	0.88
Albumin	− 0.07	0.27
Haemoglobin	− 0.03	0.65
Total bilirubin	− 0.13	0.03^*^
Total GFR	− 0.20	<0.001^**^

*Abbreviations*: *T1DM* type 1 diabetes mellitus, *BMI* body mass index, *SBP* systolic blood pressure, *DBP* diastolic blood pressure, *WHR* waist-to-hip ratio, *FPG* fasting plasma glucose, *2hPG* 2 h postprandial glucose, *HbA1c* glycated haemoglobin A1c, *GA* glycated albumin, *Cr* creatinine, *CRP* C-reactive protein, *TC* total cholesterol, *TG* triglycerides, *HDL-C* high-density lipoprotein cholesterol, *LDL-C* low-density lipoprotein cholesterol, *GFR* glomerular filtration rate

^*^ *P* < 0.05, ^**^ *P* < 0.01

**Table 3:** Multiple stepwise regression analysis showing the variables independently associated with 24 h urinary microalbumin

Independent variable	Standardised β	t	*P*-value
Cr	0.45	8.34	<0.001^**^
Total bilirubin	−0.19	−3.65	<0.001^**^
SBP	0.15	2.85	0.005^**^

The original model included the duration of diabetes, BMI, SBP, WHR, Cr, uric acid, CRP, TG, total bilirubin, GFR

*Abbreviations*: *BMI* body mass index, *SBP* systolic blood pressure, *WHR* waist-to-hip ratio, *Cr* creatinine, *CRP* C-reactive protein, *TG* triglycerides, *GFR* glomerular filtration rate

^**^ *P* < 0.01

### Bilirubin concentrations in serum are independently associated with diabetic nephropathy

The significant 24 h urinary microalbumin-related findings prompted a binary logistic regression analysis to identify factors that were independently correlated with DN. When DN was set as the dependent variable and bilirubin concentrations, duration of diabetes, BMI, SBP, DBP, WHR, HbA1c, GA, CRP, Fasting C peptide, FPG, CRP, HDL-C, TG, TC, LDL-C, Albumin, Haemoglobin were set as covariates, bilirubin concentrations were identified as an independent protective factor for DN (OR = 0.05, 95% confidence interval [CI]: 0.01–0.66), while duration of diabetes (OR =1.15, 95% CI: 1.07–1.22) and SBP (OR = 1.05, 95% CI: 1.02–1.08) were identified as independent risk factors for DN (all *P* < 0.01, Table 4).

**Table 4:** Independent risk factors associated with the diabetic nephropathy in patients with type 1 diabetes

	B	Wald	*P*-value	Exp (B)	95% CI
Duration of diabetes (years)	0.14	17.38	<0.001^**^	1.15	1.07–1.22
SBP (mmHg)	0.05	8.93	<0.001^**^	1.05	1.02–1.08
Total bilirubin (mg/d)	−3.01	5.17	0.002^**^	0.05	0.01–0.66

Variables of the original model included duration of diabetes, BMI, SBP, DBP, WHR, HbA1c, GA, CRP, Fasting C peptide, FPG, CRP, HDL-C, TG, TC, LDL-C, Albumin, Haemoglobin and total bilirubin. Only significant variables are presented

*Abbreviations*: *BMI* body mass index, *SBP* systolic blood pressure, *WHR* waist-to-hip ratio, *FPG* fasting plasma glucose, *HbA1c* glycated haemoglobin A1c, *GA* glycated albumin, *CRP* C-reactive protein, *TC* total cholesterol, *TG* triglycerides, *HDL-C* high-density lipoprotein cholesterol, *LDL-C* low-density lipoprotein cholesterol, *CRP* C-reactive protein, *HDL-C* high-density lipoprotein cholesterol, *TG* triglycerides, *95% CI* 95% confidence interval

^**^ *P* < 0.01

### Bilirubin concentrations in serum are negatively associated with the prevalence of DN in patients with T1DM

The prevalence of DN in patients with T1DM for each quartile of bilirubin concentrations is illustrated in Table 5. The first quartile group had the highest prevalence of DN (21.90%) among the four quartiles. From the second to the fourth quartile group, the prevalence of DN decreased from 17.10% to 2.00% as bilirubin concentrations increased. ORs from the third to fourth quartile were statistically significant compared to the first quartile using logistic regression analyses (all *P* < 0.05), but not in the second quartile (*P* = 0.48). After adjusting for duration of diabetes, BMI, TG, Albumin, Haemoglobin and WHR, the ORs for the prevalence of DN decreased significantly with the quartiles of bilirubin concentrations (*P* for the trend = 0.04). Therefore, we deduced that high bilirubin concentrations in serum (0.80–1.90 mg/dL) may be a protective factor for the development of DN in Chinese patients with T1DM.

**Table 5:** Prevalence of DN in patients with T1DM according to quartiles of serum bilirubin concentrations

Bilirubin quartile	Prevalence (%)	Unadjusted		Adjusted	
		OR (95% CI)	*P*-value	OR (95% CI)	*P-*value
Q1 (*n* = 64)	21.90	1 (Referent)			
Q2 (*n* = 76)	17.10	0.74 (0.32–1.71)	0.48	0.52 (0.19–1.41)	0.20
Q3 (*n* = 68)	7.40	0.28 (0.10–0.84)	0.02*	0.24 (0.07–0.82)	0.04*
Q4 (*n* = 50)	2.00	0.07 (0.01–0.58)	0.01*	0.11 (0.01–0.92)	0.02*
P trend for ORs			0.02*		0.04*

Adjustment based on duration of diabetes, BMI, TG, Albumin, Haemoglobin and WHR

Bilirubin quartiles: Q1, 0.20–0.60 mg/dL; Q2, 0.60–0.80 mg/dL; Q3, 0.80–1.00 mg/dL; Q4, 1.00–1.90 mg/dL

*OR* odds ratio, *95% CI* 95% confidence interval

*P < 0.05

## Discussion

Several previous studies have reported that high bilirubin concentrations in serum are negatively associated with the incidence of hypertension [16] and T2DM [17]. In addition, a recent study demonstrated that bilirubin concentrations were significantly negatively correlated with log (UAE) in patients with type 1 diabetes [18]. We performed a cross-sectional study to examine whether bilirubin concentrations in serum are associated with the prevalence of DN in patients with T1DM. We found that bilirubin concentrations were independently and negatively associated with albuminuria and the prevalence of DN in patients with T1DM. We deduced a range of bilirubin concentrations (0.80–1.90 mg/dL) that may serve as protective factors for the development of DN in Chinese patients with T1DM and likely represent a pharmacologically attractive target for slowing the development of DN.

We found that bilirubin concentrations and HbA1c level were not relevant (*r* = 0.06, *P* = 0.334) in 258 patients with type 1 diabetes mellitus (33 patients with diabetic nephropathy, 225 patients without diabetic nephropathy). However, a study from Choi SW et al. evaluated the relationship between HbA1c and bilirubin in 690 patients with type 2 diabetes mellitus and found that bilirubin concentrations were negatively associated with HbA1c, independent of gender, age, and other confounding factors [19]. Because several studies have confirmed that high bilirubin concentrations are inversely associated with insulin resistance [20–22], T1DM is possibly due to β-cell destruction and leads to absolute insulin deficiency rather than insulin resistance. Furthermore, results from Mianowska B et al. showed that serum total bilirubin concentration is an independent factor inversely associated with HbA1c level in young patients with type 1 diabetes (type 1 diabetes duration of more than 12 months, age from 2 to 18 years) [23], which means that in T1DM, the results are controversial. Thus, we infer that the unsynchronized results of relationship between serum bilirubin concentration and HbA1c in patients with diabetes mellitus were attributed to the age, duration of diabetes mellitus and glycemic control. Therefore, we admitted that further study should be designed to investigate the relationship between serum bilirubin concentration and HbA1c in type 1 diabetes mellitus.

Although several studies have been performed, the exact mechanism underlying the negative association between high bilirubin concentrations and the progression of T1DM remains unknown. A previous study demonstrated that diabetic hyperbilirubinemic Gunn j/j rats with high concentrations of unconjugated bilirubin in plasma excreted significantly less urinary albumin than diabetic non-hyperbilirubinemic Gunn j/+ rats, and that administration of biliverdin (5 mg/kg) protected against both albuminuria and renal mesangial expansion in db/db mice. The same authors subsequently discovered that treatment with bilirubin and biliverdin completely inhibited oxidative stress-induced increased expression of NOX4 mRNA and protein levels of cultured vascular endothelial and mesangial cells [8]. These findings suggest that bilirubin and biliverdin significantly inhibit NADPH-dependent superoxide production. A study using an isolated, perfused rat kidney model demonstrated that micromolar dose administration of exogenous bilirubin resulted in significantly improved renal vascular resistance, urine output, glomerular filtration rate, tubular function, and mitochondrial integrity after ischemia-reperfusion injury (IRI) and showed that bilirubin pretreatment may have future clinical applications, particularly in IRI after organ transplantation [24]. Furthermore, studies on diabetic rats have shown that exogenously administered CO or bilirubin can protect endothelial cells from oxidative stress-mediated injury [25]. A study on hyperbilirubinemia caused by atazanavir treatment in 16 subjects with T2DM indicated that hyperbilirubinemia is associated with a significant improvement in endothelial function [26]. Considering that oxidative stress, described as increased levels of reactive oxygen species, may be a common pathway linking diverse mechanisms underlying the pathogenesis of complications in diabetes [2] and that hyperglycemia-induced increases in blood pressure and changes in endothelial cells are reversed by antioxidants [27, 28], high concentrations of bilirubin in serum may potentially serve as protective factors against the development of DN through their antioxidative properties.

This study had several limitations. First, only total bilirubin concentrations in serum were measured, without distinguishing between conjugated versus unconjugated bilirubin. Second, this was a single-center, cross-sectional study, which prevented us from drawing conclusions regarding the temporal nature of the observed association between bilirubin concentrations and the prevalence of DN in Chinese patients with T1DM. Third, the study population consisted of Chinese males and females and whether our findings apply to other ethnic groups remains unclear.

## Conclusion

In conclusion, our study evaluated the association between total bilirubin and albuminuria, the prevalence of DN in Chinese patients with T1DM. Our data indicate that high bilirubin concentrations in serum may be protective factors for the development of DN.

## Abbreviations

DN: Diabetic nephropathy
GAD: Glutamate decarboxylase
GFR: Glomerular filtration rate
HO: Heine oxygenase
IHD: Ischemic heart disease
T1DM: Type 1 diabetes mellitus
